# 

**Published:** 1890-05

**Authors:** 


### CONTENTS.

### EDITORIALS.

PAGE

Medicine an Empirical Art, . . . . . . . . . . 193
Colleges, Journals, Pharmacies, . . . . . . . . . . 194
Sequelæ, . . . . . . . . . . . . . . 197
Coincidences, . . . . . . . . . . . . . 198

### MISCELLANEOUS.

The Repetition of the Dose. Dr. Ad. Lippe, . . . . . . . 199
Mark Twain's Tribute to Homœopathy. E. J. L. . . . . . 203
A Protest. T. F. Allen, M. D., . . . . . . . . . . 205
What is the Simillimum? M. A. A. Wolff, M. D., . . . . . 208
The Importance of a Diagnosis. F. S. Davis, M. D., . . . 210
Homœopathy in Pain. Rev. J. K. Mendenhall, . . . . . . 213
Reply to "A Synopsis of a Case of Hemorrhoids." E. W. Berridge, M. D., . . . . . . . . . . . . . . 215
Two Clinical Cases. Robert Farley, M. D., . . . . . . . 219
Clinical Cases. G. W. Sherbino, M. D., . . . . . . . . 219
La Grippe. E. T. Balch, M. D., . . . . . . . . . . 222
Verifications. E. T. Balch, M. D., . . . . . . . . . 223
What shall a Homœopathic Physician Read? Wm. Steinrauf, M. D., 224
American Institute, Session of 1890. Pemberton Dudley, M. D., . . 226
The Kansas City Homœopathic College. Annette G. Waggoner, M. D., 228
Notes on the Characteristics of Aloes. Walter M. James, M. D., . 229

### BOOK NOTICES AND REVIEWS.

Three Years' Experience of Water Purification by means of Iron. E. Devonshire, . . . . . . . . . . . . . 231
Consumption: Its Cause and Nature. R. R. Gregg, M. D., . . . 233
International Medical Annual and Practitioner's Index for 1890. P. W. Williams, M. D., . . . . . . . . . . 234
Transactions of Homœopathic Medical Society of New York for 1889, 235
Influenza and Common Colds. W. T. Ferrie, M. D., . . . . . 235
Every Thursday. Charles Robinson, . . . . . . . . 235
The Concordance Repertory of the More Characteristic Symptoms of the Materia Medica. Wm. D. Gentry, M. D., . . . . 236
The Treatment of Snake-Bites. Wm. B. Clarke, M. D., . . . . 236
Regulation of Travel and Traffic. Penna. State Board of Health, . 236
Homœopathic Prescription. M. A. A. Wolff, M. D., . . . . . 237
Precautions against Consumption. Penna. State Board of Health, . 237
A Corrector Corrected, . . . . . . . . . . . 238
The New Remedies, . . . . . . . . . . . . 238

### NOTES AND NOTICES.

International Hahnemannian Association; Notice of Meeting—I. H. A. An Appeal from the Bureau of Clinical Medicine—I. H. A. Bureau of Surgery—Homœopathic Medical College of Missouri—The Southern Journal of Homœopathy, . . . 238-240

